# Comparison of diffusion ranges at different local anesthetic volumes during superior laryngeal nerve block

**DOI:** 10.1186/s12871-024-02490-0

**Published:** 2024-03-19

**Authors:** Yin Bao, Huijun Wang, Lifeng Li, Hongbo Xu, Yun Li, Guyan Wang

**Affiliations:** 1 Department of Anesthesiology, Beijing Tongren Hospital, Capital Medical University, No.1 DongjiaoMinxiang, Dongcheng District, Beijing, 100730 China; 2 Department of Otolaryngology Head and Neck Surgery, Beijing Tongren Hospital, Capital Medical University, Key Laboratory of Otolaryngology Head and Neck Surgery (Ministry of Education of China), Beijing, 100730 China; 3 Department of Anesthesiology, Beijing Tiantan Hospital, Capital Medical University, No. 119, South Fourth Ring West Road, Fengtai District, Beijing, 100070 P.R. China

**Keywords:** Superior laryngeal nerve block, Local anesthetic dosage, Fresh larynx specimen, Dye spread, Ultrasound measurement

## Abstract

**Objectives:**

Ultrasound-guided superior laryngeal nerve (SLN) block is a practical and painless approach to avoid the hemodynamic stress response during endotracheal intubation and relieve sore throat after laryngeal surgery. The main purpose of this study was to establish an optimal dosage of local anesthetic when performing SLN block to help anesthetists balance analgesia and side effects.

**Methods:**

Twenty fresh larynx specimens were obtained immediately after resection and then injected with 2-, 3-, 4-, or 5- mL of a lidocaine-blue dye mixture at bilateral SLN puncture sites. Superficial areas of deposited blue dye were measured. Dye leakage and surrounding dyed tissue were recorded. Another 40 patients were included in the ultrasound investigation. Distances between the internal branch of the SLN (iSLN) and adjacent structures were calculated.

**Results:**

The dye spread area was greater with the administration of larger doses, especially to the visceral space. A 2- or 3-mL injection of local anesthetic was sufficient to infiltrate the SLN gap. A higher incidence of dye leaking out of the thyrohyoid membrane and anterior epiglottis space was observed; furthermore, there was substantially more dyed hyoid/thyroid cartilage with 4 and 5 mL of injected dye mixture than 2 mL. There was no significant difference between the specimen and ultrasound measurements of for length of iSLN-adjacent structures.

**Conclusions:**

In the Chinese population, 2- or 3- mL of local anesthetic is a safe dose during SLN block. A larger volume could overflow from the cavity to cause complications. The thyrohyoid membrane combined with the superior laryngeal artery is a reliable target for positioning the iSLN during ultrasound-guided regional anesthesia.

**Supplementary Information:**

The online version contains supplementary material available at 10.1186/s12871-024-02490-0.

## Introduction

Superior laryngeal nerve (SLN) block is optimal management to facilitate awake endotracheal intubation during dyspnea treatment and general anesthesia by inhibiting cough reflex and laryngospasm, which can provide patient safety and comfort and reduce hemodynamic fluctuations [1]. Moreover, SLN block can relieve postoperative pain in the larynx and pharynx above the glottis after laryngeal surgery [2]. However, there is no perfect SLN block regimen that can both meet clinical requirements and prevent potential complications.

The SLN arises from the ganglion nodosum, descends by the side of the pharynx accompanied by the internal carotid artery and the vagus nerve, and then divides into two branches at the surface of the hyoid bone. The internal branch of the superior laryngeal nerve (iSLN) is the main branch. It supplies sensory fibers that are distributed to the laryngeal mucosa, including the lower tongue surface, the epiglottis, and the glottis. The external branch of the superior laryngeal nerve (EBSLN) contains the motor fibers that innervate the cricothyroid muscle. When the EBSLN is blocked or injured, hoarseness, vocal fatigue, and frequency of sound wave change can occur, resulting in language communication issues and impacting a patient’s ability to work and quality of life after surgery [3]. When anesthetists perform SLN block, it is difficult to separate the two branches, which thus commonly causes complications. One reason is the variations in the anatomy of the EBSLN. Another reason is the excess injected volume of local anesthetic that diffuses to the EBSLN and adjacent cervical vagus nerves and carotid sheath which is just closed to the cervical sympathetic trunk and the recurrent laryngeal nerve to cause corresponding symptoms of nerve reflex [4, 5].

Currently, there is little evidence to guide the ideal volume of local anesthetic injected into the iSLN without spreading to the EBSLN and visceral space of the neck. Several studies have been performed to understand the anatomical variations in SLNs in Chinese adults [6]. However, most of these studies have been carried out based on autopsies. Formalin fixation causes tissue shrinkage, and the tissue space changes substantially [7]. As a result, there might be a large bias in the range of tissue marking dye diffusion in vivo. Our study was performed on fresh human larynx specimens immediately after total laryngectomy, most nearly the same as in vivo. The aim was to determine the optimal dose during SLN block to have the best anesthetic effects but the fewest side effects.

## Methods

The present study was approved by the Clinical Trial Ethics Committee of Beijing Tongren Hospital (TREC2022-KY091). Informed consents were obtained from all patients recruited during the preoperative period. The trial was registered prior to patient enrollment at the Chinese Clinical Trial Registry (ChiCTR2200066583, https://www.chictr.org.cn/showprojEN.html?proj=178475, Principal investigator: Yin Bao, Date of registration: 09/12/2022). This manuscript adheres to the applicable STROBE guidelines.

### Patients

Twenty male hypopharyngeal carcinoma or larynx carcinoma patients underwent total laryngectomy and 40 patients (20 male and 20 female) with nodules or polyps at the vocal cord underwent selective micro-direct laryngoscopic resection between December 10, 2022, and April 10, 2023 at the Beijing Tongren Hospital in Beijing, P. R. China.

Inclusion criteria: ①ASA I ~ III level; ②Age between 50 and 75-years-old; ③BMI: 22 ~ 28 kg/m^2^; ④Patients undergoing elective total laryngectomy or micro-direct laryngoscopic surgery.

Exclusion criteria: ①History of head and neck surgery; ②Head and neck chemoradiotherapy affect laryngeal structures; ③Laryngeal cancer invading important laryngeal structures which could affect data measuring; ④Intraoperative damage to important laryngeal structures; ⑤Patients who were severely obese or had the abnormal anatomical structure of the neck.

### Data collection and analysis

#### Specimen preparation and measurement

Fresh larynx specimens were obtained from 20 male hypopharyngeal carcinoma or larynx carcinoma patients from December 10, 2022, to April 10, 2023. Each side of the specimen was regarded as a separate object. All the objects were randomized into 4 groups (*n* = 10 in each group). Simulated SLN blocks were manipulated by injection of 2-, 3-, 4-, and 5- mL 2% lidocaine mixed with 0.01% methylene blue dye using a 22-gauge 50 mm blunt-tip peripheral nerve block needle (UniPlex NanoLine, PAJUNK®, Canada). The iSLN block injection technique was used on the specimen under direct visual observation. (as shown in Supplementary Fig. 1).

**Fig. 1:**
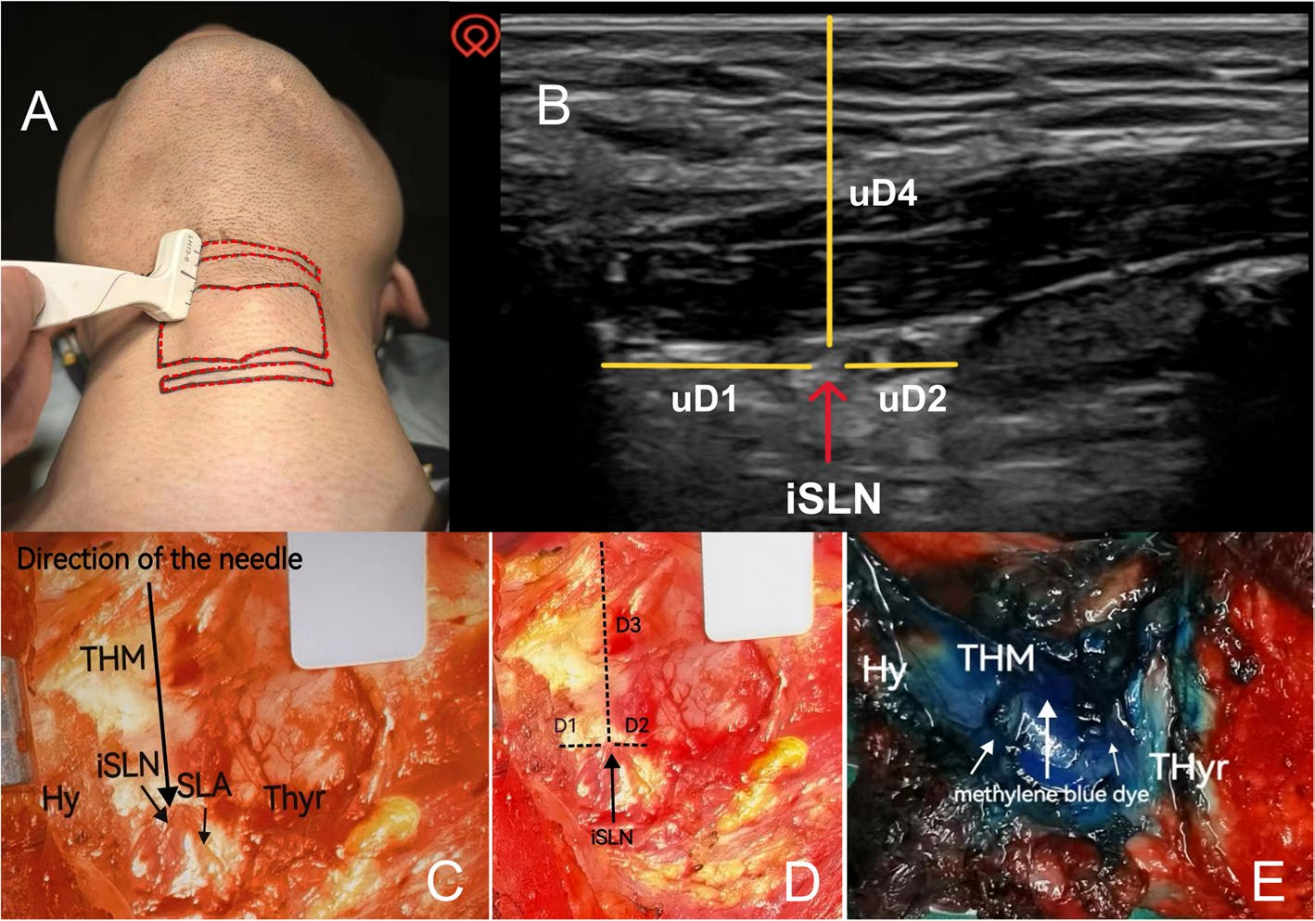
Schematic diagram illustrating anatomy and ultrasound localizations ** A** Diagram of ultrasonic probe location; **B** Schematic diagram illustrating anatomy and ultrasound localizations Measurement of SLN puncture site to surrounding structures under ultrasound; **C** Schematic diagram illustrating anatomy and ultrasound localizations Dissection of the right side of the larynx; **D** Measurement of SLN puncture site to surrounding structures on the fresh larynx specimens; **E** Photograph of the lidocaine/methylene blue mixture deposited after SLN blocking. iSLN, internal branch of the superior laryngeal nerve; THM, thyrohyoid membrane; Hy, hyoid bone, greater horn; Thyr, thyroid cartilage; SLA, superior laryngeal artery; uD1, distance between the iSLN and hyoid; uD2, distance between the iSLN and thyroid cartilage; D3, distance between the iSLN and anterior midline of the neck; uD4, the vertical distance from the skin surface

At 30 min after dye injection, macroscopic anatomical dissection was performed to expose the thyrohyoid membrane. Superficial areas of the deposited lidocaine/methylene blue mixture were measured. The occurrences of methylene blue dye overflowing from the puncture site at the thyrohyoid membrane (MOPT), penetrating the anterior epiglottis space, and staining the anterior epiglottis space, the hyoid, and the thyroid cartilage were recorded. Distances between the iSLN and hyoid (D1), thyroid cartilage (D2), and the anterior midline of the neck (D3) were measured (As shown in Fig. 1D).

#### Ultrasound-guided noninvasive measurements

Twenty male and twenty female patients with nodules or polyps at the vocal cord from December 10, 2022, to April 10, 2023, were prospectively included in our noninvasive ultrasound measurement investigation. A 6–15 MHz high-frequency linear array probe was placed over the neck area in the supine position (As shown in Fig. 1A). Sonographically, the iSLN was established lateral to the superior laryngeal artery and crossed the surface of the thyrohyoid membrane. Lengths between the location of iSLN and hyoid (uD1), thyroid cartilage (uD2), and the anterior midline of the neck (uD3), and the vertical distances from the skin surface (uD4) were measured under ultrasound scanning (Fig. 1B).

#### Statistical analysis

##### Determining the sample size

To perform a hypothesis test for this study, it was assumed that there were significant differences in the rate of MOPT among the groups. According to our pilot tests, the incidence of thyrohyoid membrane dyeing among all the dye-injected fresh specimens in the 2-, 3-, 4-, and 5- mL injection groups were 18%, 47%, 66%, and 100%, respectively. If α = 0.05 and β = 0.2 for 2-tailed testing, the sample size was calculated using the following formula: The minimal sample size for each group was 8. For a constant 20% failure rate of errors, we finally increased the sample size by 10 per group, for a total of 40.

##### Statistical tests

One-way repeated-measures ANOVA was used to compare the vertical and horizontal diameters of blue dye-stained areas on the thyrohyoid membrane among groups. Fisher’s exact test was used to compare the occurrences of MOPT, the permeated anterior epiglottis space, and the dyed anterior epiglottis space, hyoid, and thyroid cartilage. Distances between the iSLN and hyoid (D1/uD1), thyroid cartilage (D2/uD2), and the anterior midline of the neck (D3/uD3), and vertical distances from the skin surface (uD4) were assessed for normality by the Kolmogorov–Smirnov (K-S) test, and descriptive statistics were calculated using GraphPad Prism software (version 9.2.0; GraphPad Software Inc., San Diego, California, USA). Statistical significance was defined as a *P* value of less than 0.05.

## Results

Demographics of all the patients with hypopharyngeal carcinoma/larynx carcinoma and vocal cord disorders included in the study are shown in Table 1.

**Table 1:** Mean demographics of patients included in the study

	Patients with hypopharyngeal carcinoma or laryngocarcinoma	Patients with vocal cord disorders
Demographics	Mean (range); [95% CI]	
Age (years)	68 (55, 86)	46 (22, 72)
Sex	20 males	20 males and 20 females
Height (cm)	172.3 (165, 183)	166.5 (155, 182)
Weight (kg)	73.25 (62, 92)	69.23 (50, 90)
Neck circumference(cm)	43 (36, 48); 95% CI [41.69, 44.31]	44 (36, 49); 95% CI [42.80, 44.85]
Thyromental distance (cm)	7.35 (6.5, 9.0); 95% CI [7.036, 7.664]	7.49 (6.4, 9.0); 95% CI [7.310, 7.665]

By morphological anatomy, the iSLN was identified in all larynx specimens (Fig. 1C). After total laryngectomy, the EBSLN was transected, so nearly none of the EBSLN was demonstrated. The hyoid, thyroid cartilage, thyrohyoid membrane, and anterior epiglottis space were dissected for macroscopic measurements. The thyrohyoid membrane and the anterior epiglottis space were heavily stained in most of the specimens (Fig. 1E). The measurement data did not differ according to the side of the larynx specimen.

A higher ratio of MOPT with increasing injection dose was observed instantly upon injection. Furthermore, permeation of the anterior epiglottis space increased with increasing dose. As a result, there were significantly more incidences of a dyed anterior epiglottis space, hyoid, and thyroid cartilage with injections of 4- and 5- mL of blue dye than with 2 mL (*P* < 0.05), as shown in Table 2.

**Table 2:** Comparison of the incidences of adjacent tissue staining in fresh larynx specimens after iSLN puncture

	2 mL	3 mL	4 mL	5 mL
MOPT	1/10	3/10	6/10*	10/10****
*P*		0.2910	0.0286	< 0.0001
anterior epiglottis space penetrated	2/10	4/10	7/10*	10/10***
*P*		0.3142	0.0349	0.0004
anterior epiglottis space stained	3/10	4/10	10/10**	10/10**
*P*		0.50	0.0015	0.0015
hyoid dyed	0/10	6/10**	7/10**	10/10****
*P*		0.0054	0.0015	< 0.0001
thyroid cartilage dyed	4/10	6/10	9/10*	10/10**
*P*		0.3281	0.0286	0.0054

*MOPT* methylene blue dye overflowing from the puncture site at the thyrohyoid membrane

^*^P < 0.05, ^**^P < 0.01, ^***^P < 0.001, ^****^P < 0.0001 compared with the 2 mL group

**Fig. 2:**
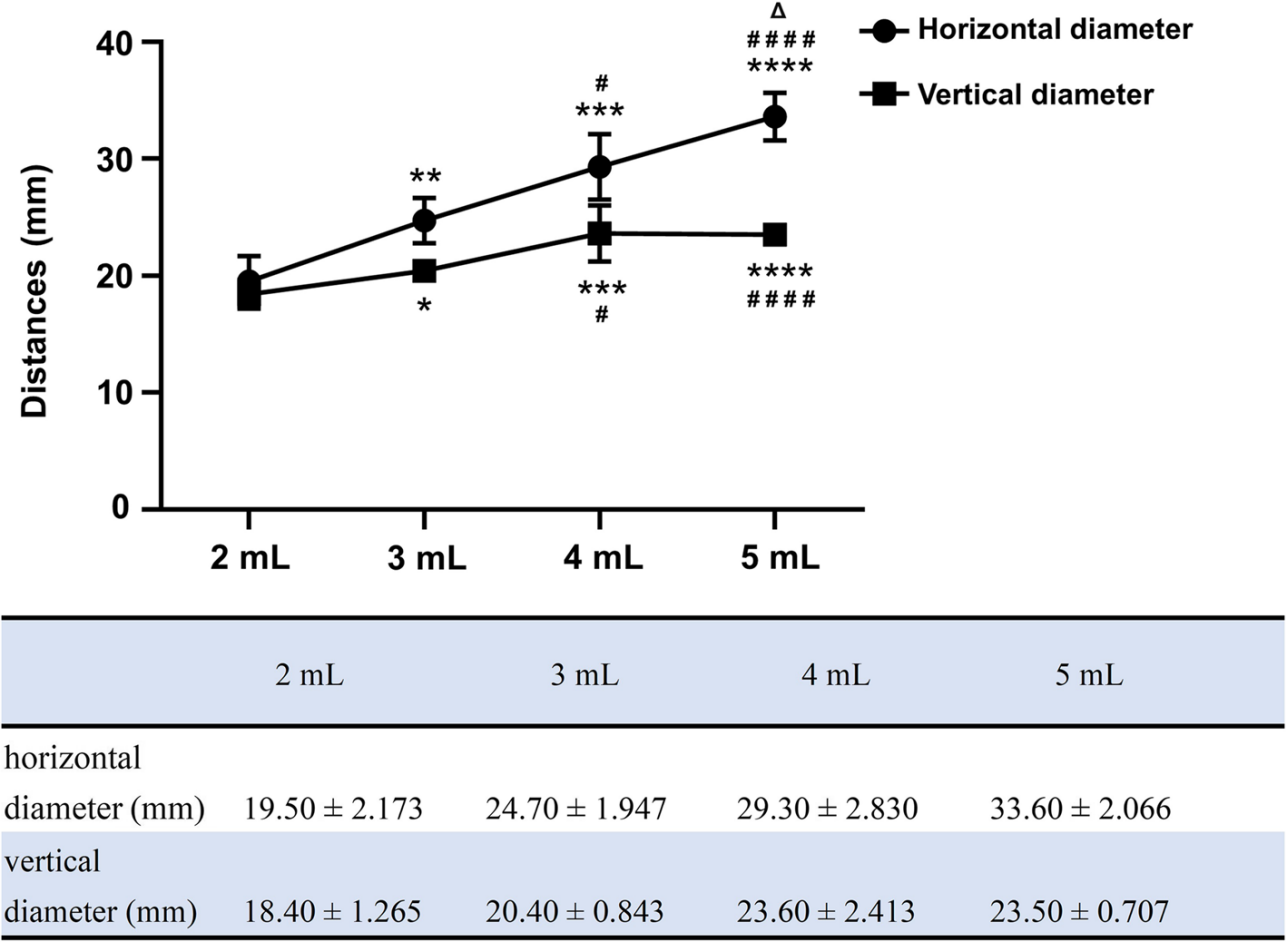
Comparison of the vertical and horizontal diameters (mm) of the blue dye deposition region of the thyrohyoid membrane among different dose groups. *P < 0.05 compared with the 2 mL group; ^#^*P* < 0.05 compared with the 3 mL group; ^Δ^*P* < 0.05 compared with the 4 mL group

After investigating the spread areas of the dye mixture solution, different blue dye staining dimensions on the surface of the thyrohyoid membrane were manifested by different volumes. Lateral diffusion increased more significantly with higher dose administration than longitudinal diffusion, as shown in Fig. 2.

**Fig. 3:**
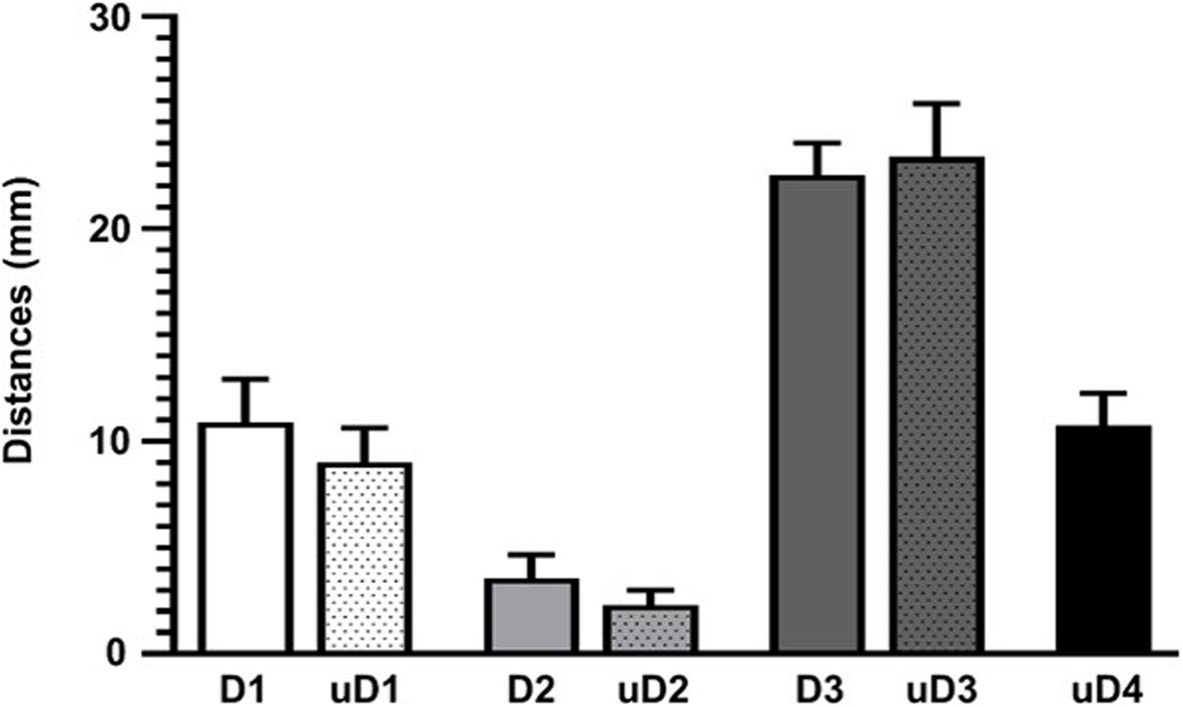
Graph exhibiting the mean interface distances from the iSLN to adjacent structures (*n* = 40). D1: Distances between iSLN and hyoid, D2: Distances between iSLN and thyroid cartilage, D3: Distances between iSLN and the anterior midline of the neck, D4: The vertical distances from the skin surface. The prefix “u” represents ultrasound-guided measurement

By measuring the specimen, the mean interface distances of iSLN—hyoid (D1), iSLN—thyroid cartilage (D2), and iSLN—anterior midline of the neck (D3) were 10.90 ± 2.040 mm, 3.55 ± 1.098 mm, and 22.50 ± 1.536 mm, respectively. For ultrasound-guided calculations, the average lengths of iSLN—hyoid (uD1), iSLN—thyroid cartilage (uD2), iSLN—anterior midline of the neck (uD3) and the depth of iSLN (uD4) were 9.02 ± 1.607 mm, 2.27 ± 0.728 mm, 23.36 ± 2.517 mm, and 10.74 ± 1.533 mm, respectively. As shown in Table 3, there was no significant difference between sexes in the average lengths of the iSLN-hyoid and iSLN-thyroid cartilage (uD1, uD2) (*P* > 0.05), but there was a large discrepancy in the average lengths of the iSLN—anterior midline of the neck and depth (uD3, uD4) (*P* < 0.05). As shown in Fig. 3, individual ultrasound measurements of D1, D2, and D3 were nearly the same as anatomical measurements on average.

**Table 3:** Comparison of sex differences in the distances between the iSLN puncture site and the adjacent structures under ultrasound-guided measurement

	uD1 (mm)	uD2 (mm)	uD3 (mm)	uD4 (mm)
male	9.41 ± 1.782	2.49 ± 0.794	25.23 ± 1.687****	11.13 ± 1.779*
female	8.63 ± 1.320	2.04 ± 0.584	21.50 ± 1.695	10.34 ± 1.127

^*^P < 0.05 compared with the female group

^****^P < 0.0001 compared with the female group

## Discussion

In the present study, we provide some evidence to help anesthetists avoid preventable complications during ultrasound-guided SLN block. It is quite unexpected that the local anesthetics dosage used in SLN blocks shouldn’t be necessarily increased as bigger as better. Even a small dose (2- or 3- mL) is enough to infiltrate the entire periphery space of the internal branch of the superior laryngeal nerve to achieve our desired effects.

For effective clinical outcomes, the best numbing target is the internal branch. The concept of the “SLN space” was proposed by Barberet et al. as an anatomical basis for iSLN block access [8]. However, the puncture space of the iSLN is a narrow cavity. Excessive local anesthetic easily spreads to neighboring nerves and tissues. A higher dose may be associated with a better peak paralytic effect but a wider range of diffusion areas which might cause a host of unwanted complications [9]. Unfortunately, few anatomical studies have been performed to establish the space size of the iSLN puncture site. The maximum safe dosage of local anesthetics needs to be determined to balance the anesthetic effects and related side effects.

Stopar-Pintaric et al. presented a worldwide ultrasound-guided iSLN block technique relying on the thyrohyoid membrane to confirm the accurate placement of the needle [10]. Although the needles penetrated the thyrohyoid membrane, liquid leaking from the puncture site could be observed even at a lower dose. As dose increased, the leakage rate increased. When the injection dose was 4- or 5- mL, the escaping liquid even spread to the visceral space of the neck. Since the SLN is a small nerve without a nerve sheath, local anesthetic cannot be enwrapped and limited. At higher doses, nearly 100% of local anesthetic spreads to the anterior epiglottis space, the hyoid, and the thyroid cartilage. This means that the nerves and muscles between these structures should be paralyzed. Under these circumstances, complications during SLN block might occur. Based on our measurement data, women might be more susceptible to some unwanted outcomes. As a result, we recommend that 2- or 3- mL of local anesthetic is safe, 5 mL is not recommended, and 4 mL can be used under close monitoring based on clinical needs, such as extending the SLN block duration.

The SLN is an important structure, and damage during thyroidectomy should be avoided. The SLN is a small nerve that is hard to visualize under direct vision and ultrasound. The anatomical landmark of the SLN is often based on surrounding bony structures. Precise body surface positioning is beneficial for surgery and anesthesia. Measurement results in patients and fresh specimens presented in the present study add an accurate data set of SLN location to the surgical anatomy database, which will help surgeons and anesthetists better trace the path of the nerve.

Ultrasound is one of the most commonly used convenient medical imaging tools in the operating room. However, the ultrasound beam deforms when it passes through different acoustic impedances. The average error has been proven to be 0.5-pixel width [11]. We have compared the anatomical lengths and ultrasound distances surrounding the SLN, and the differences are negligible. Therefore, when performing an SLN block, ultrasound-guided puncture is precise and reliable. This might be attributed to the superficial location of the SLN; as a result, the ultrasonic beam travels a short distance and has less deformation.

### Limitations

First, simulated injection was carried out on the specimens but not on actual patients. Some of the tissues were removed by the surgeon. The spreading area of local anesthetic might have a small variance to the real SLN block. Second, since men have a fivefold higher incidence of larynx cancer than women [12]. Combined with the social factors in China, female patients who underwent total laryngectomy could hardly be included. As a result, it was difficult to rule out sex differences in diffusion area studies. Nonetheless, to compensate for this defect, we established a supplementary trial to compare sex differences in ultrasound images of structures surrounding the iSLN. The results showed that there was no significant difference between sexes in the average lengths of the iSLN-hyoid and -thyroid cartilage, but male patients had larger distances than female patients of the iSLN-anterior midline of the neck and depth. That means local anesthetics are more prone to widely spread in females than male ones. For this reason, women are more susceptible to SLN block complications. It is postulated that the safe volume of local anesthetics in women is less than in men. For the sake of security, 2 mL local anesthetics used in SLN block for female patients are safer.

## Conclusions

The distances from the superior laryngeal nerve to the surrounding tissues show little variation. As a result, the body surface positioning is reliable. During blocking, 2- or 3- mL of local anesthetic is sufficient to infiltrate the iSLN fiber. Excessive amounts of local anesthetic solution might spread to remote tissues, which might cause consequent complications. The use of 4 mL or more of local anesthetic is risky. Thus, our next research direction is to attempt to add adjuvants to reduce adverse effects. Future studies are needed that focus on the paralysis effects of different doses of local anesthetics in clinical cases to balance the advantages and disadvantages during SLN block.

### Supplementary Information

**Supplementary Material 1. **

**Supplementary Material 2. **

**Supplementary Material 3. **

## Data Availability

All data generated or analyzed during this study are included in this published article.

## Abbreviations

SLN: Superior laryngeal nerve
iSLN: Internal branch of the superior laryngeal nerve
EBSLN: External branch of the superior laryngeal nerve
MOPT: Methylene blue dye overflowing from the puncture site at the thyrohyoid membrane
THM: The thyrohyoid membrane
Hy: Hyoid bone, greater horn
Thyr: Thyroid cartilage
SLA: Superior laryngeal artery
