# Supplementary material for: Comparison of diffusion ranges at different local anesthetic volumes during superior laryngeal nerve block
Source: BMC Anesthesiol. 2024 Mar 19;24:107. doi: 10.1186/s12871-024-02490-0 (PMC10949710; doi:10.1186/s12871-024-02490-0)
Supplement: Supplementary file 1 — Supplementary Material 1. [file 12871_2024_2490_MOESM1_ESM.docx]

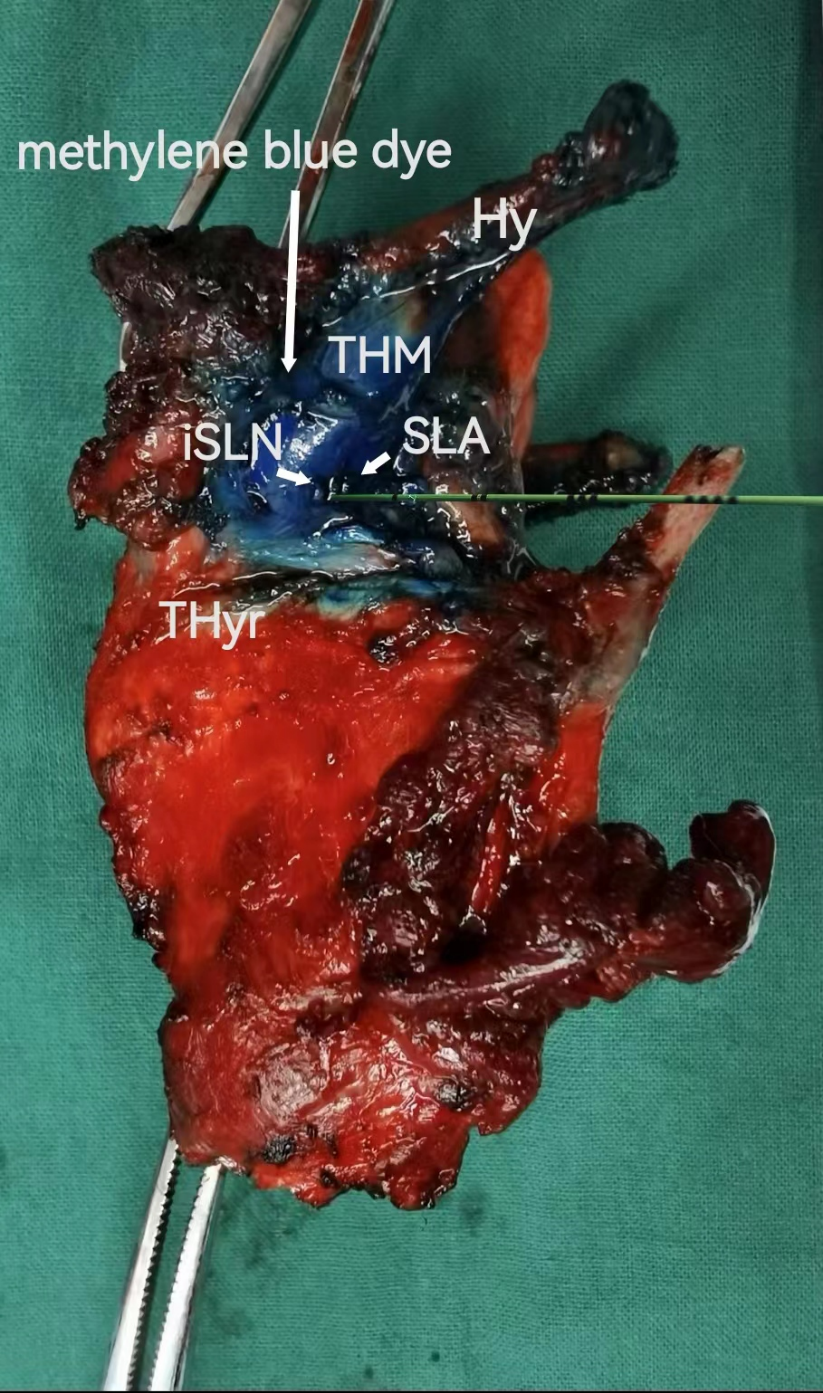


**Supplementary Figure 1** Diagram shows the simulated SLN blocking on fresh larynx specimen. A needle was injected at the site of the internal branch of the superior laryngeal nerve penetrating from the thyrohyoid membrane just next to the superior laryngeal artery under direct visual observation. iSLN, internal branch of the superior laryngeal nerve; THM, thyrohyoid membrane; Hy, hyoid bone, greater horn; THyr, thyroid cartilage; SLA, superior laryngeal artery.
